# Yeasts Occurring in Surface and Mouth Cavity of Two Chelonian Species, *Podocnemis expansa* Schweigger and *P. unifilis* Troschel (Reptilia: Chelonia: Pelomedusidae), in the Javaés River Border of Araguaia National Park in Brazil

**DOI:** 10.1155/2010/504524

**Published:** 2010-09-26

**Authors:** Paula Benevides de Morais, Raphael Sanzio Pimenta, Inara Brito Tavares, Virginia de Garcia, Carlos Augusto Rosa

**Affiliations:** ^1^Laboratório de Microbiologia Ambiental e Biotecnologia, Campus Universitário de Palmas, Universidade Federal do Tocantins, 77020220 Palmas, TO, Brazil; ^2^Laboratorio de Microbiología Aplicada y Biotecnología, Centro Regional Universitario Bariloche, Universidad Nacional del Comahue CCT-Comahue, INIBIOMA, San Carlos de Bariloche (8400), provincia de Río Negro, Brazil; ^3^Departamento de Microbiologia, ICB. Universidade Federal de Minas Gerais, 31270-901 Belo Horizonte, MG, Brazil

## Abstract

Thirty-eight specimens of free-ranging *Podocnemis expansa* (Amazon turtle) and 22 of *P. unifilis* (Tracajá) were screened for yeast isolation from surface (plastron, skin, and nails), eye, and mouth cavity. A hundred and eighteen yeast isolates belonging to 39 species were obtained. *Debaryomyces hansenii*, *Candida galli*, *C. sake*, and *Rhodotorula mucilaginosa* were the most frequent species isolated from these chelonians. Species diversity measured by Shannon's index was shown to be low and a degree of dominance could be detected as species known as potential pathogens were commonly isolated. The effective number of species in plastron of *P. expansa* was higher than in mouth samples, but not in *P. unifilis* probably due to dietary factors. *P. expansa* animals were captured on the beaches, and the superficial yeast populations may include terrestrial species. *P. unifilis* animals were captured in the water and the yeasts from superficial sites may represent species from river water.

## 1. Introduction

According to Summerbell [1], an important component of the fungal biodiversity of any given area occurs in habitats defined or conditioned primarily by vertebrates. Such habitats include the animals themselves which are colonized by commensals and disease-causing fungi, as well as organic materials making up dwelling places of those animals. Jones et al. [2] have studied fungi occurring in fecal samples of the Eastern box turtle (*Terrapene carolina carolina*), a facultative mycovore reptile that may play an important role in fungal spore dispersion. These authors isolated two yeasts, *Cryptococcus albidus* and *Rhodotorula mucilaginosa*, that are reported to naturally occur on *Trifolium* seeds found in fecal samples. Pathogenic interactions are mostly opportunistic [3] and yeasts present a variety of hosts [4–9]). Kostka et al. [10] did postmortem examination in 91 reptiles that revealed that the intestines of 80.6% of the animals carried yeasts. The authors found 56 yeast isolates belonging to the genera *Candida* (39), *Trichosporon* (13), *Torulopsis* (9), and *Rhodotorula* (3), and one nonidentified teleomorph yeast species. However, they point that no sufficiently reliable criteria could be established to prove that yeasts are associated with disease in reptiles. Erosion and traumatic lesions are common in scutelum and plastron of aquatic turtles that may be caused by algae, bacteria, and fungi [11].

We studied the yeasts occurring in *Podocnemis expansa *(Amazon turtle) and *Podocnemis unifilis* (Tracajá), reptiles of Testudines family that occur in the rivers of Araguaia Plains, a wetland area protected by the Araguaia National Park, Cantão State Park and Cantão Protection Area. These two turtle species are under threat of extinction, and are extensively used as a food source by riverine human populations. They are ectothermic aquatic animals that lay down under the sun to thermoregulate, accelerate digestion, and free the body from parasites, and their diet includes plant parts, especially seeds, and a small amount of crustaceans [12]. The inventory of mycobiota found in wild specimens would possibly extend the knowledge of yeast diversity and distribution and help to elucidate pathogenic associations encountered in captive animals in conservationist farms in the region.

## 2. Materials and Methods

The region of study is localized in the Araguaia Plains, between 9°50'S and 11°10'S and 49°56'W and 50°30'W, in the surrounding area of Araguaia National Park and Bananal/Cantão Protection Area, in the State of Tocantins, Brazil. Animals were captured in the Javaés River, under the research license 081/04-IBAMA/RAN. Due to conservation policies, the number of captured individuals was limited to 100 chelonians or less. Individuals of both chelonians were captured during the period from September to October 2004 and 2005 in three collection efforts of a week in each month, in each year. *Podocnemis unifilis* individuals were captured in the shallow river waters from 11:00 to 13:00 every day using nets or direct catching. *P. expansa *individuals were captured by direct catching from 0:00 to 2:00 every night in the Javaés River beaches of Canguçu, Comprida, Coco, Goiaba and Bonita during oviposition. Thirty-eight specimens of *P. expansa* and 22 specimens of *P. unifilis *were captured. Samples were obtained by surface scratching of ventral and dorsal plastron, nails, and skin under the plastron with sterile scalpel, and the material was transferred to Mycosel Agar, incubated at room temperature for 3 to 30 days. Sterile swabs were used for samples of material from mouth cavity, inoculated in peptone-water (Merck), and taken to the laboratory, where they were spread on Mycosel Agar, and incubated to 37°C and room temperature for growth of yeasts and observed for 3 to 7 days. All the strains grown on media were described, isolated, and identified according to standard methods [13], and when two or more strains from the same sample were identified as belonging to the same species they were considered as one single isolate for purpose of calculation of occurrence and frequency. Since, the total occurrence corresponds to the total number of isolates in each sample. Identities were verified using the taxonomic keys of Kurtzman and Fell [14] and recent publications on description of new yeast species.

Genomic DNA isolation and microsatellite-PCR fingerprinting using the primer M13 (5′-GAG GGT GGC GGT TCT-3′) were performed as described by Libkind et al. [15]. This molecular technique was used for grouping the yeast isolates prior the sequencing. Identification of the prevalent yeast species was confirmed by sequencing the D1/D2 variable domains of the large subunit rRNA gene. D1/D2 divergent domains were amplified by PCR as described by Lachance et al. [16] using the primers NL-1 (5′-GCATATCAATAAGCGGAGGAAAAG-3′) and NL-4 (5′-GGTCCGTGTTTCAAGACGG-3′). The D1/D2 variable domains of the large subunit rDNA were amplified by polymerase chain reaction (PCR) from whole cells as described previously [15]. The amplified DNA was concentrated and cleaned on WizardSVcolumns (Promega, USA), and sequenced in a MegaBace 1000 automated sequencing system (Amersham Biosciences, USA). The sequences were edited with the program DNAMAN, version 4.1 (Lynnin Bio-Soft, QC, Canada) [16]. Existing sequences for type and strains of the yeast species were retrieved from GenBank.

Simpson's index (l) and Shannon's index (H9) of diversity [17] were calculated and converted to the *effective numbers of species* calculated as the exponential of Shannon's entropy that means the number of equally-common species required to give a particular value of an index. After conversion, diversity is always measured in units of number of species [18]. The frequency was considered as the proportion of individuals from one species in relation to the total number of individuals in the sample, and the constancy was considered as the percentage of samples in which one particular species was present. Both values were calculated as in Silva [19]. Constant species were considered as those present in 50% or more samples.

## 3. Results

Table 1 presents the occurrence (number of samples positive for the presence of yeasts) and frequencies (proportion of one yeast species in relation to the total number of individuals in the sample, expressed as a percentage of the total) of 121 yeast strains of 32 species among 120 samples of both chelonian species. The effective number of species in samples of chelonians from Javaés river sites was 18.1 whereas species richness was equal to 35. The molecular approach confirmed the identification of yeast species obtained from the use of taxonomic keys of Kurtzman and Fell [14]. The microsatellite-PCR fingerprinting resulted in the grouping of strains belonging to the same species, and no groups of strains from different species were obtained. 

*Debaryomyces hansenii, Candida galli, C. sake, *and *Rhodotorula mucilaginosa* were the most frequently isolated species, in frequencies higher than 10% in the 120 sampled chelonians. *D. hansenii *and* C. sake* were the only species occurring in plastron and mouth of both chelonian species. *C. galli* occurred in all sites samples except for mouth cavity of *P. unifilis*. *P. tannicola *was isolated from plastron and mouth of *P. expansa* and from mouth of *P. unifilis. Rh mucilaginosa *occurred in plastron of *P. expansa* and in plastron and mouth of *P. unifilis. C. melibiosica* was obtained only from samples of *P. expansa* whereas *C. podzolicus* was isolated only from *P. unifilis *samples. *C. maris *and *D. vanrijae* were isolated only from plastron of both chelonians. No yeast species was obtained from eye cavity and nails of the animals. 

No yeast species could be considered as a constant species associated with the chelonians, with a constant species being the one present in more than 50% of the samples [19]. Sixty eight strains belonging to 28 species were obtained from 76 samples of *P. expansa*. Ten species were represented by only one strain. Forty-four samples from *P. unifilis *presented *D. hansenii *and* C. sake *as most frequent yeasts isolated with frequencies above 10% among 53 strains from 26 species. Nine species were represented by only one strain. Thirteen yeast species were isolated solely from samples of *P. expansa *and 12 species were obtained only from samples of *P. unifilis*, usually in low frequency.

Five yeast species were isolated solely from mouth of *P. unifilis* and included *C. laurentii*, that was one of the most frequent yeasts in this turtle species. Four species were isolated solely from mouth samples of *P. expansa* and all could be considered of incidental occurrences.

The effective number of species for plastron of *P. expansa* was 12.1 and 6.6 for mouth samples. In *P. unifilis*, from which we collected 22 individuals, plastron samples resulted in 24 strains of 13 species from which four could be considered constant, and mouth samples resulted in 29 strains of 13 species from which five were constant. The effective number of species for plastron and mouth of *P. unifilis *was 9.9.

## 4. Discussion

The calculated effective number of species was lower than the actual richness of the sample, and according to Joust [18] the greater the difference between the two numbers, the greater the dominance in the sample. *Debaryomyces hansenii, C. galli, C. sake, *and *R. mucilaginosa*, the most frequently isolated species, could possibly be considered dominant in the community. Especially *D. hansenii* and *R. mucilaginosa* are usually associated with aquatic habitats and plant materials [8, 20]. *Rhodotorula mucilaginosa *is also the most common species in *Rhodotorula *fungemia and an emerging opportunistic pathogen [21]. Also *C. galli*, a species described in association with chicken breast and liver [22], is associated with the spoilage of fresh and processed poultry [23]. These two yeasts were common especially in plastron of *P. expansa*, but not in mouth samples.

*C. galli *and *R mucilaginosa *were considered as constant species in plastron of *P. expansa* whereas *D. hansenii* and *P. tannicola* were considered as constant species in mouth of the same chelonian.* C. galli, C. sake, D. hansenii*, and* P. guilliermondii* were constant species in plastron of *P. unifilis*. *C. sake, Cr. laurentii, D. hansenii, P. tannicola*, and* R. mucilaginosa* were constant in samples of mouth cavity from *P. unifilis*. Solely nine among 32 yeast species were common to both chelonian species, showing a low degree of similarity of the mycobiota probably due to the different habitats where they were caught. According to Salera Jr. et al. [24], reproductive behaviour of both species is dependent on flooding cycle of the rivers and occurs during dry season, in August for *P. unifilis *and in September for *P. expansa.* The higher frequency of isolation of yeasts from plastron than mouth samples of *P. expansa *is probably related to the long periods of sunbathing and lack of feeding of females during the sampling period that coincided with reproductive season of this species. Samples of *P. unifilis* showed similar yeast frequencies in mouth and plastron samples probably because its reproductive period had already passed when the samplings were done, and the chelonians would have regained their feeding habits. 

More than 150 species of yeast had been associated with human pathologies [25], although Hazen [26] and Pfaller et al. [27] confirm that *C. albicans*, *C. glabrata*, *C. parapsilosis*, *C. tropicalis*, *C. lusitaniae*, *C. krusei*, *C. guilliermondii*, *C. dubliniensis*, and *Cryptococcus neoformans* remain the most prevalent yeast species encountered in clinical isolates from human sources. Among these latter species, only *C. parapsilosis *was isolated and in low frequency of occurrence, indicating that the yeast biota from chelonians may not represent an important health risk to human populations that use these animals as food, pets, and raw materials for handicrafts. 

The absence of isolation of yeasts from eyes and nails and also of dematiaceous and dermatophytic fungi does not indicate potential fungal pathogenesis in these animals, although they are reported as common in terrestrial reptiles [28–30]. Paré et al. [31] report that only one isolate of keratinollitic fungi was obtained from 127 squamate reptiles, showing that these fungi are probably not part of the mycobiota of healthy captive reptiles. 

We could hypothesize that our results may correlate with the period and the different strategy of capture of the chelonians. It is known that these chelonian species stop feeding in the reproductive period [24], and we captured *P. expansa* female individuals during oviposition activity whereas the individuals of *P. unifilis* were males and females captured in the river, probably during feeding.

## Figures and Tables

**Table 1:** Occurrence and frequencies of yeast species in surfaces (plastron) and mouth of *Podocnemis expansa *and* P. unifilis. *

Yeast species	*P. expansa*		*P. unifilis*		Total
	Plastron (*n* = 38)^1^	Mouth (*n* = 38)	Plastron (*n* = 22)	Mouth (*n* = 22)	(*n* = 120)
*Candida boidinii*	1^2^ (2)^3^				1 (0,8)
*C. galli*	10 (20)	1 (5,5)	3 (12,5)		14 (11,5)
*C. guilliermondii*	1 (2)				1 (0,8)
*C. maris*	1 (2)		1 (4)		2 (1,6)
*C. melibiosica*	1 (2)	2 (11)			3 (2,4)
*C. palmioleophila*		1 (5,5)			1 (0,8)
*C. parapsilosis*				2 (6,6)	2 (1,6)
*C. sake*	4 (8)	1 (5,5)	3 (12,5)	5 (16,6)	13 (10,6)
*C. silvicultrix*		2 (11)			2 (1,6)
*C. tenuis*	1 (2)				1 (0,8)
*C. tepae*		1 (5,5)			1 (0,8)
*Cryptococcus albidus*				1 (3,3)	1 (0,8)
*C. laurentii*				4 (13)	4 (3,2)
*C. podzolicus*			1 (4)	2 (6,6)	3 (2,4)
*Debaryomyces hansenii*	1 (2)	6 (33)	5 (20,8)	5 (16,6)	17 (13,9)
*D. vanrijae*	1 (2)		1 (4)		2 (1,6)
*D. occidentalis *var.*occidentalis *		1 (5,5)			1 (0,8)
*Filobasidium floriforme*				1 (3,3)	1 (0,8)
*Kluyveromyces wickerhamii*			1 (4)		1 (0,8)
*Pichia anomala*	3 (6)				3 (2,4)
*P. guilliermondii*	5 (10)		4	1 (3,3)	10 (8)
*P. inositovora*			1 (4)		1 (0,8)
*P. membranifaciens*				1 (3,3)	1 (0,8)
*P. pastoris*	1 (2)				1 (0,8)
*P. sydowiorum*			1 (4)		1 (0,8)
*P. tannicola*	3 (6)	3 (16,6)		3 (10)	9 (7,3)
*P. wickerhamii*				1 (3,3)	1 (0,8)
*Prototheca moriformis*	1 (2)				1 (0,8)
*P. * *ulmea*	2				2 (1,6)
*Pseudozyma aphidis*	1 (2)				1 (0,8)
*Pseudozyma prolifica*	1 (2)				1 (0,8)
*Rhodotorula acuta*			1 (4)		1 (0,8)
*R. mucilaginosa*	10 (20)		1 (4)	3 (10)	14 (11,5)
*R. ulmea*	2 (4)			1 (3,3)	3 (2,4)
*Trichosporon asahii*			1 (4)		1 (0,8)

TOTAL	50	18	24	29	121

^1^Number of samples. ^2^Number of samples positive for the presence of the yeast species. ^3^Frequency is expressed in percentage as the proportion of one yeast species in relation to the total number of individuals in the sample: *p*_*i*_ = *n*_*i*_/*N*, where *n*_*i*_: number of individuals of species *i* and *N*: total number of individuals.
